# Mutational Evolution of *Pseudomonas aeruginosa* Resistance to Ribosome-Targeting Antibiotics

**DOI:** 10.3389/fgene.2018.00451

**Published:** 2018-10-18

**Authors:** Fernando Sanz-García, Sara Hernando-Amado, José L. Martínez

**Affiliations:** Centro Nacional de Biotecnología, Consejo Superior de Investigaciones Científicas, Madrid, Spain

**Keywords:** antibiotic resistance, *Pseudomonas aeruginosa*, tobramycin, tigecycline, mutation, evolution

## Abstract

The present work examines the evolutionary trajectories of replicate *Pseudomonas aeruginosa* cultures in presence of the ribosome-targeting antibiotics tobramycin and tigecycline. It is known that large number of mutations across different genes – and therefore a large number of potential pathways – may be involved in resistance to any single antibiotic. Thus, evolution toward resistance might, to a large degree, rely on stochasticity, which might preclude the use of predictive strategies for fighting antibiotic resistance. However, the present results show that *P. aeruginosa* populations evolving in parallel in the presence of antibiotics (either tobramycin or tigecycline) follow a set of trajectories that present common elements. In addition, the pattern of resistance mutations involved include common elements for these two ribosome-targeting antimicrobials. This indicates that mutational evolution toward resistance (and perhaps other properties) is to a certain degree deterministic and, consequently, predictable. These findings are of interest, not just for *P. aeruginosa*, but in understanding the general rules involved in the evolution of antibiotic resistance also. In addition, the results indicate that bacteria can evolve toward higher levels of resistance to antibiotics against which they are considered to be intrinsically resistant, as tigecycline in the case of *P. aeruginosa* and that this may confer cross-resistance to other antibiotics of therapeutic value. Our results are particularly relevant in the case of patients under empiric treatment with tigecycline, which frequently suffer *P. aeruginosa* superinfections.

## Introduction

Antibiotic resistance has been a major public health concern since the dawn of the antibiotic era, but in recent decades there has been an alarming increase in the number and type of antibiotic-resistant bacteria (Appelbaum, 2012), posing a threat to health worldwide (Roca et al., 2015). Predicting the mechanisms by which bacteria may acquire resistance is therefore important in the prevention and treatment of infections (Martinez et al., 2007; Martinez et al., 2011).

Resistance can be acquired via horizontal transfer of antibiotic resistance genes (HGT), or through mutation (Hernando-Amado et al., 2017). While exhaustive information is available on the mechanisms of antibiotic resistance at the basic science and epidemiological levels, the evolutionary trajectories leading to high level antimicrobial resistance, as well as the reproducibility of these trajectories among populations evolving concurrently, have been studied in less detail. It is worth mentioning, however, that the reconstruction of mutants that are selected in patientsunder treatment have shown that fitness costs and the selection of compensatory mutations are critical for the success of some specific antibiotic resistance mutations (Shcherbakov et al., 2010; Brandis et al., 2015; Meftahi et al., 2016; Huseby et al., 2017). Nevertheless, this type of retrospective analyses is useful just for studying already known mechanisms of resistance, not for predicting new ones (Pietsch et al., 2017).

Strategies to predict the emergence of resistance mutations (Martinez et al., 2007) were implemented soon after the discovery of antibiotics (Bryson and Szybalski, 1952), one of the most useful of which is experimental evolution. Since the seminal work of Richard Lenski, experimental evolution has been used to analyze different bacterial traits, including the development of resistance to antibiotics (Bryson and Szybalski, 1952; Toprak et al., 2011; Turrientes et al., 2013; Feng et al., 2016; Ibacache-Quiroga et al., 2018). Recent research has shown experimental evolution able to predict the emergence of resistance against different antimicrobial agents, including colistin (Jochumsen et al., 2016), beta-lactams, quinolones and aminoglycosides (Cabot et al., 2016; Feng et al., 2016; Ibacache-Quiroga et al., 2018; Lopez-Causape et al., 2018b). In recent years, the potential of experimental evolution has been further boosted by the development of technologies that allow the fast and affordable sequencing of whole bacterial genomes.

*Pseudomonas aeruginosa*, an opportunistic pathogen widely distributed in nature (Silby et al., 2011), commonly causes lung, airway and other infections in hospitalized patients. It is the main cause of chronic infections in patients with cystic fibrosis (CF) and chronic obstructive pulmonary disease (Martinez-Solano et al., 2008; Tummler et al., 2014). These infections are usually fought using aminoglycosides, β-lactams and polymyxins (Palmer and Whiteley, 2015). Unfortunately, *P. aeruginosa* intrinsically shows low-level susceptibility to a number of drugs, even against the recently developed glycylcycline tigecycline (Pankey, 2005), which works via tightly binding to the ribosome and thus evading the most common tetracycline resistance mechanisms. In addition, mutants presenting increased levels of antibiotic resistance are selected along chronic infections. Indeed, while resistance to aminoglycosides in *P. aeruginosa* isolates from acute infections has been largely attributed to the acquisition of antibiotic resistance genes, mutation plays a major role for the acquisition of resistance by *P. aeruginosa* causing chronic infections (Vogne et al., 2004; Guenard et al., 2014; Bolard et al., 2018).

In the present work, experimental evolution and whole-genome sequencing (WGS) were used to examine evolutionary trajectories of *P. aeruginosa* toward resistance against two ribosome-targeting antimicrobials: tobramycin and tigecycline. The aim was to determine whether the mechanisms that impair the actions of different drugs targeting the same cell machinery are shared (at least in part), or whether resistance to each drug is specific via a different mechanism. Tigecycline binds to the 30S ribosomal subunit, thereby blocking the interaction of aminoacyl-tRNA with the A site of the ribosome, whereas tobramycin prevents the formation of the 70S complex (Kotra et al., 2000). While tobramycin forms part of usual therapy regimens against *P. aeruginosa* (Cheer et al., 2003), the pathogen is intrinsically resistant to tigecycline (following the clinical definition of antibiotic resistance) (Martinez et al., 2015). One cause of this phenotype is the capability of the multidrug efflux pump MexXY, also involved in intrinsic *P. aeruginosa* tobramycin resistance (Westbrock-Wadman et al., 1999), to extrude tigecycline (Dean et al., 2003). Nonetheless, tigecycline was used in the present study since it provides an opportunity to examine whether or not bacterial pathogens can acquire clinically relevant characteristics when challenged with the antibiotics to which they are considered to be intrinsically resistant. In this regard, it is worth mentioning that *P. aeruginosa* has emerged as a major cause of superinfection in nosocomial patients treated with tigecycline (Garcia-Cabrera et al., 2010; Ulu-Kilic et al., 2015; Katsiari et al., 2016). Knowing whether or not the empirical use of tigecycline for treating Gram-negative hospital infections might challenge *P. aeruginosa*, affecting its susceptibility to other antibiotics commonly used for treating *P. aeruginosa* infections, is of relevance for developing a rational approach for treating such superinfections. The results showed that even for microorganisms dubbed intrinsically resistant to an antibiotic, the challenge with this antibiotic selects mutants presenting reduced susceptibility to antibiotics of clinical value.

Experimental evolution studies allow one to determine whether evolutionary trajectories are reproducible, i.e., whether the process of evolution is mainly deterministic and hence predictable (Martinez et al., 2007), or whether it is largely stochastic. The present work provides a predictive analysis of the potential mutational causes of resistance in *P. aeruginosa* against two ribosome-targeting antibiotics belonging to different structural families, as well as the different evolutionary trajectories taken toward this resistance. This information may allow new strategies to be designed for predicting, managing, and eventually reducing resistance in this important nosocomial pathogen. In addition, the results throw light on whether bacterial evolution is largely stochastic or presents some deterministic features, and thus whether the emergence and spread of antibiotic resistance can be predicted to a certain extent (Martinez et al., 2007, 2011).

## Materials and Methods

### Growth Conditions and Determination of Susceptibility to Antibiotics

Unless otherwise stated, bacteria were grown in Mueller Hinton Broth (MHB, Pronadisa) at 37°C with agitation at 250 rpm. The initial concentrations of tigecycline (Pfizer) and tobramycin (Normon, S. L) that inhibit the growth of *P. aeruginosa* PA14 under the culture conditions used in the evolution experiments were determined at 37°C.

General susceptibility to a wide range of antibiotics - tigecycline, tetracycline, aztreonam, ceftazidime, imipenem, ciprofloxacin, levofloxacin, norfloxacin, tobramycin, streptomycin, amikacin, colistin, polymyxin B, chloramphenicol, fosfomycin and erythromycin – was examined by disk diffusion in Mueller Hinton Agar (MHA) (Sigma) at 37°C.

The MICs of different antibiotics were determined for the bacterial populations over the evolution period at 37°C in MHA using *E*-test strips (MIC Test Strip, Liofilchem^®^). MICs of colistin and polymyxin B were determined in MHB II by double dilution in microtiter plates. The MICs to the antibiotics of selection and to fosfomycin were repeated twice and in all cases, the results were the same in the replicated assays.

### Experimental Evolution

Twelve independent bacterial populations (four controls without antibiotics, four populations challenged with tigecycline, and four populations challenged with tobramycin) were grown in parallel in MHB for 35 consecutive days. All replicates were established from a stock culture of the *P. aeruginosa* PA14 strain. Each day, the cultures were diluted (1/250) with fresh MHB: 8 μl of bacterial culture in 2 ml of medium. The concentrations of tigecycline and tobramycin used for selection increased over the evolution period from the initial MIC up to 32MIC, doubling them every 5 days. Every 5 days, samples from each culture were taken and preserved at -80°C for future investigation.

### Whole-Genome Sequencing

Genomic DNA was extracted at the end of the evolution assays from all 12 populations using the Gnome^®^ DNA kit (MP Biomedicals). Whole-genome sequencing was performed by Sistemas Genómicos S.L. Libraries were obtained without amplification following Illumina protocols and recommendations. The quality of the extracted material was analyzed via a 4200 Tapestation High Sensitivity assay, and the DNA concentration determined by real-time PCR using a LightCycler 480 device (Roche). The pool of libraries was pair-end sequenced (100 × 2) in an Illumina HiSeq 2500 sequencer. The average number of reads per sample was 8646177, which represents a coverage of 200x on average. Short reads used in this publication are deposited in SRA database^1^ with accession PRJNA490803.

### Bioinformatic Analysis

Mutations in the evolved bacteria were detected using CLC Genomics Workbench 9.0 (QIAGEN) software. WGS data were trimmed and the reads aligned with the *P. aeruginosa* UCBPP-PA14 reference chromosome (NC_008463.1). The single nucleotide polymorphisms (SNPs) present in the populations kept under selective pressure were identified and filtered against those present in the populations maintained in the absence of such pressure. The cut-off threshold of a mutation to be included in the analysis was ≥15%.

### Confirmation of SNPs

Sanger sequencing was used to verify the mutations found via WGS (**Supplementary Table S1**) and to ascertain the order of appearance of these modifications. Twenty-four pairs of primers, which amplified 100–200 base pair regions containing each putative mutation, were designed (**Supplementary Table S2**). After PCR amplification, the corresponding amplicons were purified using the QIAquick PCR Purification Kit (QIAGEN) and sequenced at GATC Biotech.

### RNA Extraction and Real-Time RT-PCR

One flask containing 20 ml of MHB was inoculated with an overnight culture of the selected clones and *P. aeruginosa* PA14 to a final O.D._600_ = 0.01, and they were incubated until exponential phase was reached (O.D._600_ = 0.6). 10 ml of each culture were centrifuged at 7000 rpm for 15 min and at 4°C. This process was performed with three independent biological replicates.

Then, RNeasy mini Kit (QIAGEN) extraction protocol was followed: 570 μl of TE buffer (10 mM Tris-HCl, 1 mM EDTA [pH 8.0]) and 30 μl of lysozyme (Sigma), for a final concentration of 1 mg/ml of the latter, were added to each sample. Afterward, the samples were mixed by vortexing for 10 s and were incubated at room temperature for 10 min with regular vortexing. A volume of 2100 μl of buffer RLT (QIAGEN) was added, and samples were sonicated at 0.45 Hz for 20 s. Next, 1410 μl of ethanol (Merck) was added and the protocol continued according to the manufacturer’s instructions. In order to remove any residual DNA, two DNase treatments were carried out, with DNase I (QIAGEN) and TURBO DNase (Ambion). Finally, a PCR with *rplU* primers (**Supplementary Table S2**) was performed to check that no residual DNA was present in the RNA samples. High Capacity cDNA Reverse Transcription Kit (Applied Biosystems) was used to synthesize cDNA from 5 μg of RNA. Then, real-time RT-PCR was performed using 50 ng of cDNA and the Power SYBR green PCR Master Mix (Applied Biosystems) in the ABI PRISM 7500 real-time PCR system (Applied Biosystems). Gene expression data were normalized using the gene *rplU* (**Supplementary Table S2**). Differences in the relative amounts of mRNA were obtained following the 2^-ΔΔCt^ method (Livak and Schmittgen, 2001).

## Results and Discussion

Different publications have shown that the genes that contribute to antibiotic resistance (intrinsic resistome) comprise around 3% of the genome of a given bacterial species (Breidenstein et al., 2008; Fajardo et al., 2008; Tamae et al., 2008; Alvarez-Ortega et al., 2010; Blake and O’Neill, 2013; Fernandez et al., 2013). In the case of *P. aeruginosa* the search of a comprehensive transposon-tagged library has shown that the inactivation of 135 genes renders low-level tobramycin resistance (Schurek et al., 2008). Even if we do not take into consideration gain of function mutations (which are not considered in transposon insertion libraries studies), neither that different mutations might be selectable in each of these genes (Martinez and Baquero, 2000), hence increasing the number of potential antibiotic resistance mutants (Lopez-Causape et al., 2018a), the number of possible combinations of these 135 genes is 2.7E230. It can be argued that the number of mutations that a single bacterium can accumulate is likely low. However, even if only five mutations are accumulated as reported in the present work (see below), the number of combinations of these 135 potential mutations, taken five by five, is 4.1E10 if the order of selection is taken into consideration and 3.4E8 if the order of selection of each of the mutations along evolution is not taken into consideration. If all possible combinations were equivalent in terms of antibiotic resistance, these numbers would mean that mutation-driven antibiotic resistance should be un-predictable.

### Stepwise Evolution of *P. aeruginosa* Toward Antibiotic Resistance

To determine whether similar potential evolutionary trajectories are followed by different populations, four biological replicates were allowed to evolve in parallel under selective pressure from tobramycin (populations 1–4), tigecycline (populations 5–8) and in the absence of any selective pressure (populations 9–12). In populations 1–8 the antibiotic concentration was doubled every 5 days, from 0.5 μg/ml for tobramycin and 4 μg/ml for tigecycline, up to 32MIC. At 64MIC no growth was seen after 5 days incubation, suggesting this concentration to be beyond limits of *P. aeruginosa* when evolving toward tobramycin and tigecycline resistance in these experimental conditions.

**FIGURE 1:**
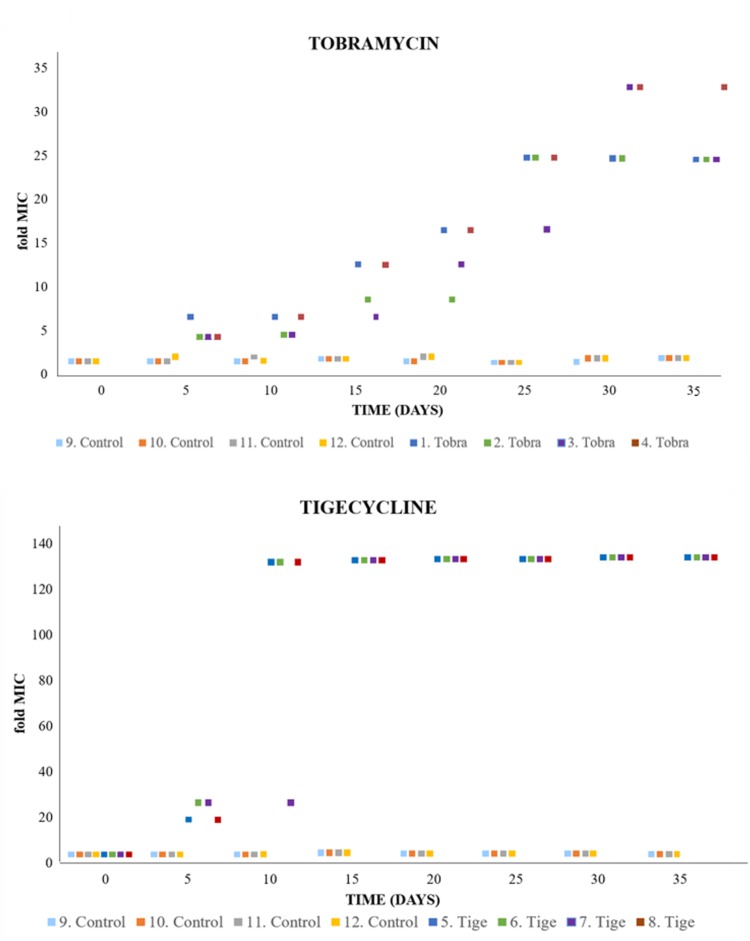
Evolution of *P. aeruginosa* under antibiotic selective pressure. Graphs show the rise of the MICs over the evolution period, from the value corresponding to the wild-type strain (tobramycin [Tobra], 1 μg/ml; tigecycline [Tige], 2 μg/ml) to high levels of tobramycin/tigecycline resistance (doubling the antibiotic concentration every 5 days). The detection limit of the tigecycline *E*-test was 256 μg/ml, limiting the assessment of resistance levels from day 15 to the end of the experiment. MIC values for each replicate are provided in **Supplementary Table S3**.

To monitor the evolution of resistance over the selection period, the susceptibility of each population to the selecting antibiotic was determined every 5 days by *E*-test. When bacteria are confronted to increased concentrations of antibiotics, two different phenotypic trajectories can be foreseen. Sudden selection of high-level resistance at the first steps of evolution or stepwise selection of mutants presenting increasing resistance levels. As shown in **Figure 1**, a stepwise evolutionary trajectory was observed for both antibiotics, suggesting either accumulation of sequential mutations after each evolution step (i.e., each change in antibiotic concentration), or the displacement of low-level resistance mutants by higher-level resistance ones as the selection pressure increased. It is important to note that the evolutionary trajectory was similar in all replicated experiments (**Figure 1**). This suggests the number of evolutionary trajectories possible (at least at phenotypic level) is limited, although the amount of genotypic evolutionary trajectories can be larger (Lassig et al., 2017).

An increase in the MIC of an antibiotic after experimental evolution does not, however, necessarily mean that antibiotic-resistant mutants have been selected for: resistance may be due to a phenotypic adaptation to the presence of an antibiotic rather than to mutations (Levin and Rozen, 2006; Martinez et al., 2009; Martinez and Rojo, 2011). To address this possibility, the evolved populations were sub-cultured in the absence of selection pressure (three sequential passages in MHB) and the MICs again determined. These were found not to change, indicating that the observed modifications were mainly due to the selection of stable mutants.

### Cross-Resistance and Collateral Sensitivity of Evolved Populations

To determine whether the development of resistance was specific to the selecting antibiotic or also affected susceptibility to other antimicrobials, a range of representative antibiotics was tested (beta-lactams, quinolones, tetracyclines, macrolides, aminoglycosides, polymyxins, and chloramphenicol) by disk diffusion (**Supplementary Table S4**). Despite it has been shown macrolides as azithromycin can select *mexCD-oprJ* overpressing mutants in *P. aeruginosa* biofilms (Mulet et al., 2009), no differences in susceptibility to imipenem and erythromycin were detected between the wild-type parental *P. aeruginosa* PA14 strain and the evolved populations. Whether these differences can be due to the different experimental model (biofilm or planktonic cells) or the different genetic background where mutants are selected (PAO1 or PA14) remains to be established. Nevertheless, almost every evolved population developed resistance against other antibiotics belonging to the different structural families, implying that at least some resistance mutations are not tigecycline- or tobramycin-specific (**Table 1**). Notably, in all cases the evolved populations were more susceptible to fosfomycin than the wild-type strain, and it is important to know whether this is related to the development of resistance to ribosome-targeting agents. In this regard, it is worth mentioning that *Listeria monocytogenes* is more susceptible to fosfomycin when growing intracellularly than when growing extracellularly. The reason for this lies in the overexpression of a hexose-phosphate transporter when the bacteria are grown intracellularly, which provides an entry route for this antibiotic (Scortti et al., 2006). These results indicate that the resistance to ribosome-targeting drugs may correlate with increasing susceptibility to fosfomycin, although the underlying mechanisms remain obscure. Given that these types of drug are widely used clinically, it is important to determine whether this trade-off occurs commonly, and whether the combined or sequential use of both types of antibiotic offers a better alternative to current therapies.

**Table 1:** MICs (μg/ml) of antibiotics of different structural families in the populations evolved at 32MIC tobramycin and tigecycline.

Replicate	Tgc	Tet	Atm	Caz	Cip	Tob	S	Ak	C	F	Cs	PB
**PA14**	1.5	16	1.5	1	0.094	1	16	2	24	24	1.5	0.75
**Tobramycin 32MIC**												
1	64	48	3	1.5	0.5	32	192	≥256	24	1	6	2.5
2	24	32	4	1.5	0.5	24	256	192	32	1	6	2.5
3	12	24	4	1.5	0.19	8	64	128	24	1	4	3
4	32	24	3	1.5	0.5	32	192	≥256	24	1.5	5	2.5
**Tigecycline 32MIC**												
5	≥256	192	8	4	0.75	4	96	32	128	2	2.5	1
6	≥256	192	8	3	0.5	1.5	64	16	64	1.5	2.5	1
7	≥256	≥256	6	3	0.25	2	128	16	96	1	5	2
8	≥256	192	6	2	0.75	8	192	32	128	2	5	1.5
**Controls**												
9	2	12	2	1.5	0.094	1	12	4	24	32	1.5	0.75
10	2	8	2	1	0.19	1	12	2	16	32	1.5	0.75
11	1.5	12	2	1	0.094	1	12	3	32	32	1.5	1
12	2	12	2	1.5	0.125	0.75	12	3	16	32	1.5	0.75


*Tgc, tigecycline; tet, tetracycline; atm, aztreonam; caz, ceftazidime; cip, ciprofloxacin; tob, tobramycin; s, streptomycin; ak, amikacin; c, chloramphenicol; f, fosfomycin; cs, colistin; pb, polymyxin B. All MICs were obtained by *E*-test, excepting for polymyxin B and colistin, which were analyzed via double dilution in a microtiter plate.*

### Mutations Selected in the Presence of Antibiotics

To gain insight into the genetic events associated with the development of resistance in the evolved populations, the genomes of each, as well as that of the original population (for which a frozen sample was available), were sequenced on the last day of the experiment (when the antibiotic concentration was 32MIC). Different mutations can appear by chance or because of the *P. aeruginosa* adaptation for growing in MHB. Consequently, only those mutations present in the populations evolving under antibiotic selective pressure and not in the control populations evolving in absence of selection were taken into consideration. In other words, only those mutations that are enriched (and hence have been selected) under antibiotic selective pressure were taken into consideration. Indeed, all mutant alleles selected in the presence of antibiotics and not present in the populations grown without antibiotics present always a coverage >50% and typically >90% (**Supplementary Table S1**) in the whole population, indicating that they are under positive selection when *P. aeruginosa* grows in presence of antibiotics. **Supplementary Table S1** shows the locations of all 35 confirmed genetic changes potentially associated with the development of resistance. A total of 31 single-nucleotide variants (SNVs) and 4 multi-nucleotide variants (MNVs; deletions and substitutions of various nucleotides and one transposition) were found, 31 located in genes and 4 in intergenic regions. The majority of the mutations located in genes resulted in amino acid alterations, frameshifts or stop codons. In addition, the coverage of the obtained reads was mapped to the *P. aeruginosa* genome to search for gene amplifications and deletions. One 377 bp deletion was detected in the tigecycline-treated population 8, comprising part of the gene coding for the transcriptional repressor of the multidrug efflux pump *mexCD-oprJ*, *nfxB*, plus a small region of the adjacent gene *morA*.

To further verify the presence in the evolved populations of the mutations identified by WGS (**Supplementary Table S1**), the regions containing these mutations were amplified and the amplicons Sanger-sequenced.

### Common Aspects of the Evolution of *P. aeruginosa* Toward Resistance to Tobramycin or Tigecycline

The present results shed light on the development of resistance to ribosome-targeting antibiotics in *P. aeruginosa*. Although this opportunistic pathogen is already intrinsically resistant to tigecycline, highly resistant populations with MICs several times higher than that seen for the wild-type strain were selected for in the experimental evolution assays (**Figure 1**).

**FIGURE 2:**
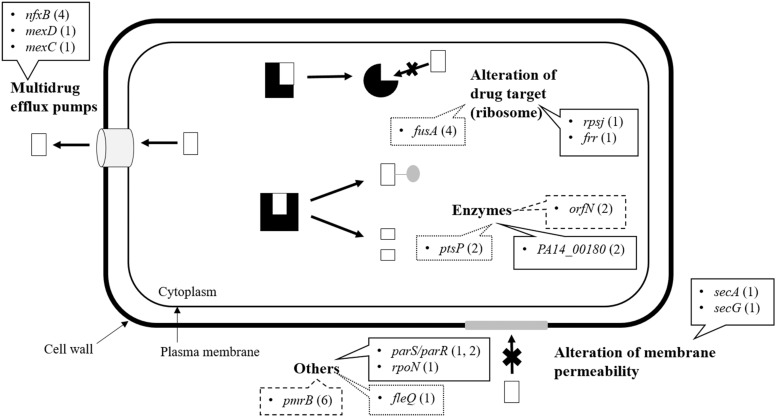
Common and different genetic changes identified by whole-genome sequencing. Mutations detected by Illumina and verified by Sanger Sequencing in evolved *P. aeruginosa* PA14 at 32MIC tigecycline/tobramycin. Most mutations fell into the four major functional groups of resistance mechanism: multidrug efflux pumps, alteration of drug target, enzyme neutralization, and alteration of membrane permeability. Others that did not properly match any of these categories were entered the group “Others.” The line type indicates the drug providing the selection pressure: continuous line, tigecycline; dots, tobramycin; dashes, both treatments. The figures after the genes refer to the number of different genetic changes found in each. These values also inform about the number of biological replicates in which a particular gene mutated, with the exceptions of *rpsJ* (only one type of mutation) and *orfN* (two different mutations that appeared in every evolved population).

Overall, the results indicate that increased resistance to tigecycline and tobramycin comes about via distinguishable evolutionary trajectories but which show some similarities (**Table 2**). In particular, mutations in *orfN* (selected in all eight replicates) and *pmrB* (selected in 6 out of 8 replicates) were selected under pressure from either antibiotic (**Figure 2**). It should be noted that all populations evolved in the presence of antibiotic, and none of those evolved in unexposed control populations show mutants in *orfN* during the first step in evolution (i.e., after the first change in antibiotic concentration). This gene codes for a putative glycosyl transferase needed for the glycosylation of type A flagellins (Schirm et al., 2004). Six of the studied mutants carried a single base pair deletion in a poly-G repeat in *orfN*, and two contained a single base pair insertion in the same region (**Supplementary Table S1**), all of them leading to a Val50fs mutation. Similar mutations have been found in *P. aeruginosa* when exposed to ciprofloxacin under experimental evolution conditions (Wong et al., 2012). The fact that these mutations are located in a poly-G repeat region supports that this gene might have a specific high mutation rate as the consequence of polymerase slippage (Moxon et al., 2006). We hypothesize that this might be the reason why *orfN* mutants are detected in all replicates.

**Table 2:** Previously described role on antibiotic resistance of genes presenting mutations in the evolved populations.

Gene name	Tobramycin replicates	Tigecycline replicates	Previously described role in resistance	References
*orfN*	4	4	Ciprofloxacin	Wong et al., 2012
*pmrB*	4	2	Cationic peptides, quinolones, tigecycline, tobramycin	Muller et al., 2011; Moskowitz et al., 2012; Lopez-Causape et al., 2018b
*fusA*	4	–	Aminoglycosides	Wang et al., 2015; Feng et al., 2016; Bolard et al., 2018; Lopez-Causape et al., 2018b
*ptsP*	2	–	Tobramycin	Schurek et al., 2008
*fleQ*	1	–	–	–
*nfxB*	–	4	Quinolones, tetracyclines, β-lactams, chloramphenicol	Masuda et al., 2000b
*mexCD*	–	2; 1 *mexC*, 1 *mexD*	Quinolones, tetracyclines, β-lactams, chloramphenicol	Masuda et al., 2000b
*PA14_00180*	–	2	–	–
*rpsj*	–	2	Tetracycline, tigecycline	Hu et al., 2005; Ahn et al., 2016
*parRS*	–	3; 2 *parR*, 1 *parS*	Cationic peptides, aminoglycosides	Fernández et al., 2010; Muller et al., 2011
*secAG*	–	2; 1 *secA*, 1 *secG*	–	–
*frr*	–	1	–	–
*rpoN*	–	1	Carbenicillin, quinolones, tobramycin	Viducic et al., 2007, 2016, 2017


*pmrAB* is a two-component system involved in polymyxin resistance (Moskowitz et al., 2012). The system regulates the expression of operons involved in the biosynthesis of lipid A with 4-aminoarabinose, which produces a more positively charged lipopolysaccharide, thus reducing the binding and the activity of cationic peptides (Gunn et al., 2000). Gain-of-function mutants with lipid A modifications can be selected for and are resistant to colistin (Fernández et al., 2010). Moreover, a recent evolution study of *P. aeruginosa* involving colistin gave rise to the selection of mutations in *pmrB* (Jochumsen et al., 2016), and it has been proposed that mutations in such regulatory elements may potentiate the effect of other mutations (Lind et al., 2015). Indeed, *pmrB* is involved in a wide range of antibiotic resistances, including those to quinolones, tigecycline, tobramycin and cationic peptides (Muller et al., 2011; Lopez-Causape et al., 2018b). In agreement with these data, the populations selected in our evolution experiment present a reduced susceptibility to colistin and polymixin B (**Table 1**).

### Evolutionary Pathways Toward Tobramycin Resistance in *P. aeruginosa*

Having shown the common features of the evolutionary trajectories toward resistance to both test antibiotics, the specific pathways toward resistance to each were sought. Mutations in *fusA* and *ptsP* were found to be involved in evolution toward tobramycin resistance (Schurek et al., 2008; Wang et al., 2015; Feng et al., 2016). Together with the above mentioned *orfN* mutants, mutations in *fusA*, which codes for elongation factor G, may be envisaged as a first response to aminoglycosides (Wang et al., 2015; Feng et al., 2016) since they were observed in all evolved populations (**Figure 2**). Further, recent works have shown this type of mutations to be present in clinical isolates as well as in *in vitro* selected aminoglycoside resistant mutants (Bolard et al., 2018; Ibacache-Quiroga et al., 2018; Lopez-Causape et al., 2018b), reinforcing its role in the acquisition of aminoglycosides resistance. Although *mexXY* overexpression is considered a hallmark of aminoglycoside resistance development of CF chronic infections (Guenard et al., 2014), our data using PA14, as well as a recent experimental study using PAO1 (Lopez-Causape et al., 2018b), show that this does not necessarily always occurs, at least *in vitro*.

**FIGURE 3:**
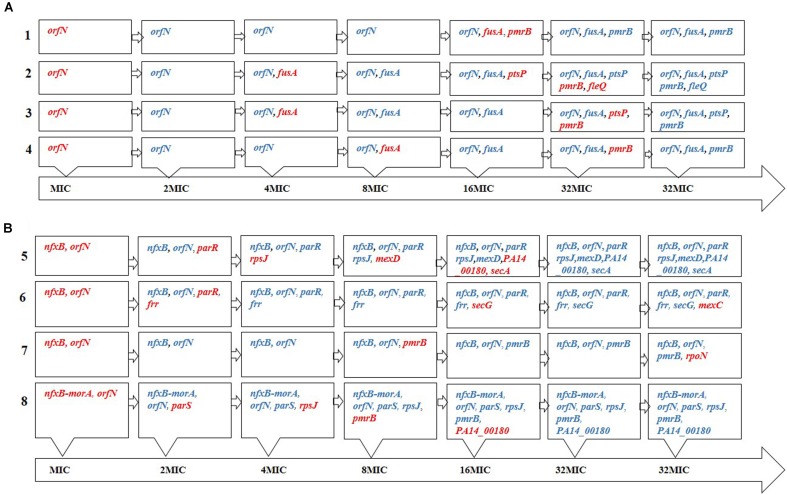
Order of appearance of genetic changes. Order of appearance of **(A)** tobramycin and **(B)** tigecycline resistance mutations during the evolution process, as determined by PCR amplifications of known SNVs/MNVs in 32MIC populations. The names of the genes in red indicate that these mutations appeared in this step. Once a mutation appears it remains in the population until the end of the evolutionary period. We cannot discard that other mutations may have appeared and not fixed over the 35 days evolution period.

Consistent with a proposed role of *ptsP* in low-level tobramycin resistance (Schurek et al., 2008), the present results indicate that its mutation might also be important, because of its presence in 2 out of 4 tobramycin replicates. In fact, the MIC clearly increased when this mutation appeared alone (population 2, 8–24 μg/ml; **Figure 3** and **Supplementary Table S3**). Whether or not this type of mutation is selected for in clinical settings deserves further investigation. Finally, FleQ is a major flagellar regulator that has been found responsive to c-di-GMP and plays a role in biofilm formation (Hickman and Harwood, 2008; Jimenez et al., 2012; Matsuyama et al., 2016), although it has not been previously described to be involved in antibiotic resistance in planktonic cells.

It has been previously shown that mutations in at least 135 genes can render low-level aminoglycoside resistance in *P. aeruginosa* (Schurek et al., 2008). Despite the large number of possible combinations that might be selected along evolution under antibiotic selective pressure, we found that all four evolved populations contained mutants in *orfN*, *fusA* and *pmrB* and two of these evolved populations also presented mutants in *ptsP*. Further, recent work performed independently in another laboratory has also shown that *fusA* and *pmrB* mutants are frequently selected in presence of aminoglycosides (Lopez-Causape et al., 2018b). Taking into consideration that the number of possible combinations of five mutations from the above mentioned 135 genes exceeds 3.4E8, our results indicate that *P. aeruginosa* mutation-driven resistance to tobramycin displays a certain degree of predictability.

### Evolutionary Pathways Toward Tigecycline Resistance in *P. aeruginosa*

*Pseudomonas aeruginosa* PA14 evolving under selective pressure from tigecycline accumulated resistance mutations, indicating that, even in intrinsically resistant microorganisms, resistance may increase. Among the selected mutated genes, three – *rpsJ, parRS* and *nfxB* - have already been described involved in antibiotic resistance (a result that validates the present experimental strategy). The Val57 change in the 30S ribosomal protein S10 (*rpsJ*), which was present in two out of four replicates, is commonly seen in tetracycline-resistant clinical isolates of pathogens such as *Neisseria gonorrhoeae*, (Hu et al., 2005) while in *Klebsiella pneumoniae* different mutations in this gene have been related to tigecycline resistance (Ahn et al., 2016). Further, the two-component *parRS* system has been extensively examined in association with resistance to various drugs. In our experimental evolution assay, three out of the four tigecycline evolved populations presented mutations in this two-component system (**Figure 3**), which may be indicative of its importance in resistance. ParRS may promote multidrug resistance via three mechanisms: by inducing the expression of efflux pumps (such as MexXY-OprM), by repressing the expression of porins, and via LPS alteration (Muller et al., 2011).

**FIGURE 4:**
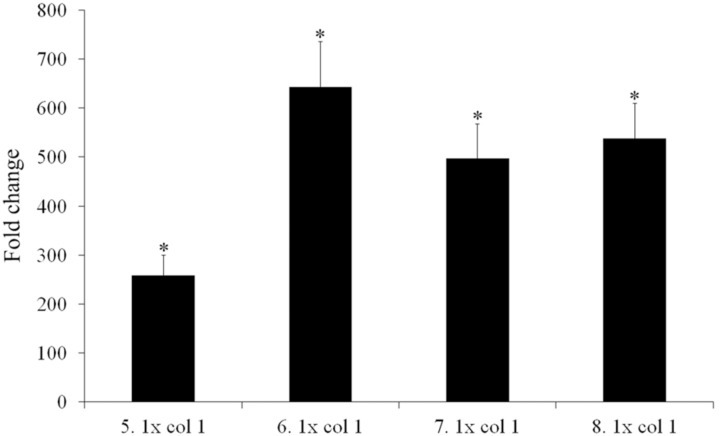
*mexC* over-expression in tigecycline evolved individual clones. Four individual clones from the tigecycline evolved populations after 5 days of the experiment were isolated. The presence of the different *nfxB* mutations, as well as the absence of *orfN* mutations, were addressed. Fold changes were estimated with respect to the value given by *P. aeruginosa* PA14 strain. Error bars indicate standard deviations of the results from three independent experiments. Statistically significant differences (*p* < 0.05) in the level of expression of each population with respect to the wild type strain was evaluated using the Student’s *T*-test and are highlighted with asterisks. 5.1–8.1: clones isolated from the populations evolved under tigecycline selective pressure at day five of evolution.

The transcriptional repressor of the multidrug efflux pump MexCD-OprJ (Purssell and Poole, 2013), one of the most clinically important of all efflux pumps in *P. aeruginosa*, is coded for by *nfxB*. Loss-of-function mutations in this gene result in the pump’s overexpression. Different mutations in *nfxB* were selected in all four replicates of the evolving populations, and most were fully inactivating mutations (a 377 bp deletion leading to a truncated protein; a transposition; and an 11 bp deletion). Although it has been suggested that the main efflux pump contributing to tigecycline resistance in *P. aeruginosa* is MexXY-OprM (Masuda et al., 2000a; Dean et al., 2003), the first step along the path to resistance in the present work was seen to be the selection of mutants able to extrude tigecycline via MexCD-OprJ. To ascertain whether or not *nfxB* selected mutations inactivate the repressor, hence allowing MexCD overexpression, one individual clone from each 5 days tigecycline evolved population was isolated in MHA. The presence of their corresponding *nfxB* mutations and the absence of *orfN* modification were confirmed by Sanger-sequencing. Expression of *mexC* was measured in comparison with the one of the wild-type strain. As shown in **Figure 4**, all clones carrying *nfxB* mutations overexpressed *mexC*, confirming that these mutations inactivate, in different degree, the NfxB repressor. Two further mutations in each of the genes coding for the subunits of this efflux pump (*mexC* and *mexD*) were then selected for in populations 5 and 6 respectively. It may be that these mutations alter the specificity of MexCD-OprJ improving the capacity of the pump to extrude tigecycline. The MexD SNV (Phe608Cys) is located in one of the two large periplasmic loops known to be involved in substrate specificity (Elkins and Nikaido, 2002). In agreement with this, it has been described that mutations in AcrB, the *Enterobacteriaceae* ortholog of MexD, alter the pump’s substrate profile (Blair et al., 2015). While a role for these mutations in reducing the susceptibility to tigecycline is a compelling hypothesis, it cannot be ruled out that mutations in *mexD* or *mexC* may compensate for any increased non-physiological extrusion of some important cellular metabolite by those mutants that overexpress this pump.

The present results indicate that tigecycline resistance might also come about through changes in the Sec system (*SecA* and *SecG*), likely via the modification of the permeability of the membrane and the impairment of tigecycline uptake. It has also been suggested that the Sec pathway could be involved in the translocation of components of multidrug efflux pumps across the bacterial membranes (Yoneyama et al., 2010; Akiba et al., 2013; Chaudhary et al., 2015), but it is unclear how changes in this translocation could increase resistance in the selected mutants. Tigecycline increased resistance may also come about through modifications of the σ_54_ RpoN factor that has been found to modulate *P. aeruginosa* virulence (Kazmierczak et al., 2005), bacterial tolerance to carbapenems (Viducic et al., 2016), susceptibility to quinolones (Viducic et al., 2007) and survival in the presence of tobramycin (Viducic et al., 2017). Both the Sec pathway and RpoN have been proposed as excellent targets in the search of novel antibiotics (Jin et al., 2016; Lloyd et al., 2017). Our findings suggest that tigecyline might select mutants presenting cross-resistance to these potential inhibitors, still under development. Mutations in the gene encoding the ribosome recycling factor *frr* are likely related to modifications in the tigecycline target (ribosome). Indeed, this particular mutation is located in the start codon (**Supplementary Table S1**), so it may affect the level of *frr*, impairing the steady state amount of active ribosomes. A similar situation might arise with *PA14_00180*, which codes for a putative rRNA small subunit methyltransferase, hence likely able to modify the ribosome.

All the changes seen during the evolution of the populations subjected to selective pressure from tigecycline indicate that evolutionary pathways toward tigecycline resistance present some common features. All four replicates present mutations in *orfN* and *nfxB* and mutations in *parRS* were selected in three out of the four populations. Mutations at *secAG, PA14_00180*, *rpsJ* or *mexCD* were selected in half (two) of the populations. This indicates that *P. aeruginosa* can develop resistance to tigecycline by following a limited number of different evolutionary trajectories, which share some specific type of mutations, suggesting a certain degree of determinism.

In addition of conferring resistance to the selecting antibiotics, the respective mutants also showed resistance to antibiotics belonging to other structural categories and with different targets. This might be explained, at least in part, via the important role that efflux pumps seem to play in the development of resistance. Indeed, all mutants selected in the presence of tigecycline showed mutations in *nfxB*, which would lead to the overexpression of MexCD-OprJ. In turn this might reduce susceptibility to quinolones, tetracyclines, beta-lactams and chloramphenicol (De Kievit et al., 2001) – phenotypes in agreement with the present data (**Table 1**). Moreover, all four population replicates analyzed showed reduced susceptibility to aminoglycosides. This phenotype may be explained in three populations as a result of the mutations selected for in the ParRS system, which is involved in modification of the lipopolysaccharide and in the regulation of the expression of MexXY-OprM, an efflux pump that contributes to aminoglycoside resistance in *P. aeruginosa* (Masuda et al., 2000a), and which is described as being regulated by the two-component system ParRS (Muller et al., 2011).

## Conclusion

Experimental evolution approaches may allow determining basic aspects of evolution, among which knowing to what extent evolution (in our case of asexual organisms) can be predictable and hence deterministic or is basically stochastic (non-predictable) (Lassig et al., 2017), is notably interesting. This is particularly relevant in the case of antibiotic resistance, a field in which the implementation of novel therapeutic approaches is based in the assumption that evolution of antibiotic resistance can be largely predictable (Martinez et al., 2007), a feature that goes against most common views on evolution.

The present work provides information on the evolutionary trajectories leading to resistance to antibiotics belonging to different structural families but targeting the ribosome in *P. aeruginosa.* The results suggest that, although mutations in several genes may contribute to the development of antibiotic resistance (Fajardo et al., 2008; Tamae et al., 2008; Alvarez-Ortega et al., 2011; Martinez, 2012; Vestergaard et al., 2016; Lopez-Causape et al., 2018a), which may imply a large degree of stochasticity in the evolutionary trajectories, mutation-driven evolution toward resistance is partially deterministic, at least when bacteria grow in the same conditions. This opens up the possibility of predicting the appearance of antibiotic resistance (Martinez et al., 2007).

The selection of specific mutations depends primarily on the fitness of each mutant before/after selection and the rate of mutation supply for each of the mutations (Huseby et al., 2017; Hughes and Andersson, 2017). Recent work has shown that a high generalized mutation supply does not alter the types of mutations selected upon experimental evolution (Ibacache-Quiroga et al., 2018), in which case fitness costs, together with population bottlenecks (Vogwill et al., 2016), will be the main constrains for selecting some mutants over other mutations able of providing the same resistance phenotype. However, the situation might be different in the case of gene-specific high mutation rates, a situation that might have happened in the case of *orfN*. To note here that all mutations in *orfN* have been selected in a poly-G region, present in the *P. aeruginosa* PA14 strain, a situation that might increase the mutation rate as the consequence of polymerase slippage (Moxon et al., 2006). We hypothesize that this gene-specific high mutational supply might be in the basis of the presence of *orfN* mutants in all replicates. However, OrfN sequence is highly polymorphic in *P. aeruginosa* (Arora et al., 2004) becoming doubtful if these results can be extrapolated to strains with different *orfN* alleles.

The present results clearly show that challenging *P. aeruginosa* with tigecycline has clinically relevant consequences, despite this bacterial pathogen is considered to be intrinsically resistant to this antibiotic. Tigecycline is used for empiric treatment of Gram-negative infections, and in this type of patients, the main agent causing superinfection is *P. aeruginosa* (Garcia-Cabrera et al., 2010; Ulu-Kilic et al., 2015; Katsiari et al., 2016), which is most likely under tigecycline selection during treatment. Our results indicate that tigecycline selects mutants presenting reduced susceptibility to antibiotics of clinical value as aztreonam, ceftazidime, ciprofloxacin or aminoglycosides. Notably, these mutants also display an increased susceptibility to fosfomycin. Our results suggest that fosfomycin might be an antibiotic of choice for treating superinfections by *P. aeruginosa* in tigecycline treated patients. A recent work has shown that most antibiotic resistance mutations display strain-independent phenotypes (Knopp and Andersson, 2018). Nevertheless, it is also true that the current work has been performed with a single, model strain and, because of the diversity of *P. aeruginosa*, it would be important to examine additional strains to establish whether this phenotype depends on the genomic context or can be extrapolated to other *P. aeruginosa* clinical isolates.

Antibiotic resistance can be acquired by the acquisition of antibiotic resistance genes, which usually confer resistance to members of the same family of antibiotics. However, particularly relevant in the case of chronic infections is the selection of antibiotic resistant mutants. Our results indicate that antibiotic resistance mutations frequently have a pleiotropic effect, altering susceptibility to other drugs. In agreement with this is the fact that some of the mutations selected for by exposure to tobramycin or tigecycline have previously been described to confer resistance to other antibiotics belonging to different structural families.

## Author Contributions

FS-G and SH-A performed the experiments. JM and SH-A designed the work. All the authors participated in the interpretation of the results and in writing the article.

## Conflict of Interest Statement

The authors declare that the research was conducted in the absence of any commercial or financial relationships that could be construed as a potential conflict of interest.

## Footnotes

1 https://www.ncbi.nlm.nih.gov/sra
