# Supplementary material for: Mutational Evolution of Pseudomonas aeruginosa Resistance to Ribosome-Targeting Antibiotics
Source: Front Genet. 2018 Oct 18;9:451. doi: 10.3389/fgene.2018.00451 (PMC6200844; doi:10.3389/fgene.2018.00451)
Supplement: Supplementary file 1 [file Data_Sheet_1.pdf]

Supplementary Table S1 | Mutations detected in tigecycline/tobramycin evolved *P. aeruginosa* PA14 populations at the end of the evolution.

Gene	Replicate	Mutation	Nucleotide localization	Aa Change	Coverage* (%)
			**BOTH TREATMENTS**		
orfN	4, 8	Ins G	138-139	Val50fs	80/61,64
	1, 2, 3, 5, 6, 7	Del G	139	Val50fs	68,90/70,98/ 87,41/84,84/ 83,68/85,55
pmrB	1	C-->A	772	Leu258Met	98,11
	2	T-->G	22	Ser8Ala	89,22
	3	G-->C	853	Val285Leu	83
	4	T-->C	110	Leu37Pro	77,59
	7	C-->T	28	Arg10Cys	98,29
	8	G-->T	583	Val195Leu	99,53
			**TIGECYCLINE TREATMENT**		
PA14_00180	5	C-->A	146	Arg49Leu	96,68
	8	C-->T	752	Cys251Tyr	100
rpsj	5, 8	G-->C	169	Val57Leu	100/98,97
parR	5	G-->A	259	Glu87Lys	98,51
	6	G-->A	640	Glu214Lys	98,36
secA	5	G-->A	1475	Ala492Val	98,41
mexD	5	A-->C	1823	Phe608Cys	99,51
nfxB	5	T-->C	452	Leu151Pro	98,04
	6	Del CTGAAAGA ACT	76	Leu26fs	53
	7	Del GGAGGC	231-236	Glu78_Ala79	82,85
	7	Ins GGAGGC	394-395	Lys132delinsArg ArgGln	98
	8	Del C	494	Ser166fs	92,85
frr	6	G-->A	3	Met1Ile	100
mexC	6	C-->A	311	Ser104*	84,46

secG	6	Del A	194	Phe65fs	93,21
rpoN	7	G-->C	64	Ala22Pro	100
parS	8	C-->G	553	Arg185Gly	100
**TOBRAMYCIN TREATMENT**					
fleQ	2	T-->G	721	Thr241Pro	53,19
ptsP	3	Del G	2156	Glu677fs	97,73
	2	G-->T	2029	Asp720fs	99
fusA	1	A-->G	2011	Thr671Ala	99,42
	2	G-->C	1783	Ala595Pro	100
	3	G-->A	1634	Gly545Asp	100
	4	C-->T	2038	Arg680Cys	99,31
**INTERGENIC MUTATIONS**					
**UPS: TrmH family RNA methyltransferase** / **DNS: *rpsF***	1, 3, 5, 7, 8	Del C	5809306	-	84,75/83,99/ 84,11/83,75/ 79,27
**UPS: hypothetical protein** / **DNS: cAMP binding protein A**	2	G-->T	5556124	-	44,38
**UPS: *tyrZ*** / **DNS: 16S ribosomal RNA**	6	GC-->AT	732714..732 715	-	89,29
**UPS: *tyrZ*** / **DNS: 16S ribosomal RNA**	6	T-->C	732732	-	92,31

The Table shows the nucleotide location of the mutations and associated amino acid changes. Genetic modifications in intergenic regions are also included, and their locations refer to the nucleotide position in *P. aeruginosa* UCBPP-PA14 reference chromosome (NC_008463.1). Fs: frameshift. UPS: upstream gene. DNS: downstream gene. Del: deletion. Ins: insertion.

* Coverage represent the percentage of reads of each mutant allele among the total number of reads, corresponding the same region in the genome, within the whole population, at the end of the experimental evolution.

Supplementary Table S2 | Primers used to verify nucleotide modification detected in whole-genome sequencing and to perform real-time RT-PCR.

Gene	Mutation	Localization	Primer Fw (5'-3´)	Primer Rv (5´-3´)
		**WGS VERIFICATION PRIMERS**		
orfN	Ins G	138-139	ATGGACGTTCCCAATGCCCG	CCGCCAGAATCAGCAAAACC
	Del G	139		
pmrB	C-->A	772	GCCGAACGCCGACTGACCAG	AATTGCTCCAGCAGGGCGTC
	G-->C	853		
	T-->G	22	CCTGAAAACCGCCTACCGGA	TTCGGTGGCAAGGTCGAGCA
	T-->C	110		
	C-->T	28		
	G-->T	583	GTTGCTCGGCGGCCTGGTCT	TGAGTTCGTCGATGAGGCCG
PA14_00180	C-->A	146	TTGCCGCCGCAACTGGACAA	AACGGCTTCTGCAACAGGCG
	C-->T	752	CAACTGGCCGCCGAGCTGCT	CCGCTGGAGGTTTTCCCGAA
rpsj	G-->C	169	GACCCGCAAGGAACGTTTCA	GGACGATGTCCAGTACACGC
parR	G-->A	259	CGCGAACCTGCCGATCCTCATA	GCTCGATCGGCTTGATCACG
	G-->A	640	TCGACGTCTGCATCAGCAAG	AACAGGTAGCCCTTGCCCCA
secA	G-->A	1475	GAGGCAGGCATCGAGCACAA	ATGTTGGTGGCGATGGTCAC
mexD	A-->C	1823	GAACTCGAGCGCTTCCTCAA	CAGTCCTTGAAGGTCGGGAA
nfxB	T-->C	452	TCCTACCTGGAAGCGCTGGA	CATCTGCTCCAGGGTATGCG
	Ins GGAGGC	394-395		
	Del CTGAAAGAACT	76	CTCATCAAGGCGCTGGCAGT	ATCTGCACCAGGTTGTCCCG
	Del GGAGGC	231-236	GAACCAGATCATCCAGGCCT	CGGTGGGTGAGGTGTTCCTT
frr	G-->A	3	TCTTGCCCATGCGCTCCTGC	TCTTGCCCATGCGCTCCTGC
mexC	C-->A	311	TTCCAGATCGATCCGGCACC	CTGCGCCTCGAACAGCACCG
secG	Del A	194	GCTACCTTTTTGAGTCGGATT	TCAGACTTTTCTTTAGCGAA
rpoN	G-->C	64	CGCTAGTCCTCAAGATGGGC	ATTTCCTGCTGGAGGTCCAG
parS	C-->G	553	ACCGTGCTGGCCTACATCCT	TCGAAGGACAGGCGGGAGAT

fleQ	T-->G	721	CTTCACCGGTGCCATCACCA	AGCAGGGCGATGTCTTCCAC
ptsP	Del G	2156	TGCTGCATGCGTTGAAGAAG	AGCGAGCTGTGGATGACCTG
	G-->T	2029		
fusA	A-->G	2011	TATTCGTGCCGAGGTTCCGC	GGAGCTTCGGCGTATTTGGA
	G-->T	2038		
	G-->C	1783	GGACGAGAAGGGCAACATCA	CATGATCGGCTCGAGCACCT
	G-->A	1634		
	T-->C	457		
**REAL-TIME RT-PCR PRIMERS**				
rplU	-	-	CGCAGTGATTGTTACCGGTG	AGGCCTGAATGCCGGTGATC
mexC	-	-	GACCTGCTGTTCCAGATCG	AGGACTTCGATACCGCCAC

Supplementary Table S3 | MIC values in the population replicates during selective pressure from tigecycline and tobramycin.

Treatment	Replicate	5 d MIC	10 d 2MIC	15 d 4MIC	20 d 8MIC	25d 16MIC	30 d 32MIC	35 d 32MIC
TOBRAMYCIN	**1**	6	6	12	16	24	24	24
	**2**	4	4	8	8	24	24	24
	**3**	4	4	6	12	16	32	32
	**4**	4	6	12	16	24	32	32
TIGECYCLINE	**5**	32	32/≥256	96/≥256	≥256	≥256	≥256	≥256
	**6**	48	24/≥256	64/≥256	64/≥256	≥256	≥256	≥256
	**7**	48	32/≥256	64/≥256	96/≥256	≥256	≥256	≥256
	**8**	32	24/≥256	64/≥256	96/≥256	≥256	≥256	≥256
CONTROLS (no antibiotic)	**9**	1-2	1-2	1.5-2	1-2	1-2	1-2	1.5-2
	**10**	1-2	1-2	1.5-2	1-2	1-2	1.5-2	1.5-2
	**11**	1-2	1.5-2	1.5-2	1.5-2	1-2	1.5-2	1.5-2
	**12**	1.5-2	1-2	1.5-2	1.5-2	1-2	1.5-2	1.5-2

The table shows the E-test MIC value for each replicate population every five days (the antibiotic concentration was doubled every 5 days). The MICs for the tobramycin and tigecycline controls are indicated as two different values separated by a dash (X-X). Double inhibition halos detected in mixed populations are shown as two MIC values separated by a slash (X/X).

Supplementary Table S4 | Disc diffusion assays of antibiotics of different structural families in the populations evolved at 32MIC tobramycin and tigecycline

Replicate	Tgc	Tet	Imi	Atm	Caz	Cip	Tob	S	Ak	Cs	PB	C	E	F
**PA14**	11	20	26	24	23	34	21	14	21	17	14	11	8	15
							**TOBRAMYCIN**							
**1**	0	7/16	28	25	19	27	7	0	0	17	15	14	0	25
**2**	0	8/19	27	25	20	31	7	0	7	16	14	15	6	26
**3**	0	9/18	28	23	20	33	13	7	12	17	14	14	8	24
**4**	0	8/17	24	22	20	27	7	0	0	16	14	15	0	26
							**TIGECYCLINE**							
**5**	0	0	26	17	18	26	16	6	15	18	14	8	6	24
**6**	0	0	26	19	15	26	18	7	19	18	14	8	6	24
**7**	0	0	27	20	18	30	19	6	18	16	14	8	6	26
**8**	0	0	24	20	20	25	14	0	14	17	14	8	6	18
							**CONTROLS**							
**9**	10	18	27	26	21	32	19	12	21	17	13	11	7	16
**10**	12	19	26	26	23	33	19	13	23	17	13	12	8	15
**11**	12	20	27	25	22	33	19	14	21	17	13	13	8	17
**12**	10	19	27	25	24	34	20	13	22	17	14	11	7	15

The table shows the diameter of halo (mm) in the disc diffusion assays for each replicate population at the end of the experimental evolution. The antibiotics in which halos differ were selected for an E-test assay (Table 1). Double inhibition halos detected in mixed populations are shown as two values separated by a slash (X/X). Tgc: tigecycline, tet: tetracycline, atm: aztreonam, caz: ceftazidime, cip: ciprofloxacin, tob: tobramycin, s: streptomycin, ak: amikacin, cs: colistin, PB: polymyxin B, c: chloramphenicol, e: erythromycin, f: fosfomycin.
